# Risk Assessment of Potentially Toxic Elements (PTEs) Pollution at a Rural Industrial Wasteland in an Abandoned Metallurgy Factory in North China

**DOI:** 10.3390/ijerph15010085

**Published:** 2018-01-06

**Authors:** Zheng Sun, Jiajun Chen

**Affiliations:** Key Laboratory for Water and Sediment Sciences of Ministry of Education, School of Environment, Beijing Normal University, Beijing 100875, China; sunjon@126.com

**Keywords:** rural industrial wasteland, potential toxic elements, pollution indices, risk assessment

## Abstract

The potential toxic elements (PTEs) pollution problems in many rural industrial wastelands have been observed to be conspicuous. Therefore, 40 top soil samples were collected from the wasteland of a typical rural metallurgy factory in Baoding, China. The total concentrations of six key PTEs were measured. The soil properties and speciation of the PTEs were also identified. Extremely high concentrations of As, Cd, Pb, and Zn were observed in the surface soils. Using the PTEs concentration in the top soils of the rural industrial wasteland, the following indices of pollution were calculated: the pollution load index (PLI), the geo-accumulation Index (I_geo_), the risk assessment code (RAC), and the health risk assessment (HRA). The analysis of the PLI and I_geo_ indicated that site #1 was relatively clean, while sites #2 and #3 were heavily polluted. The results of the RAC showed that PTEs in top soils at sites #2 and #3 were significantly increased (*p*  <  0.05) for Cd and Zn. The HRA indicated that both As and Pb presented non-carcinogenic risks to children and adults at sites #2 and #3. Our findings can be a reference for risk prevention of industrially abandoned land in rural China.

## 1. Introduction

Soils are generally regarded as a sink of potential toxic elements (PTEs) in terrestrial ecosystems. PTEs are one of the thorniest types of pollutants due to their broad distribution, hard degradation, and biological enrichment along the food chain [1]. The excessive emission of PTEs in soils may cause environmental pollution through surface runoff and infiltration and may be harmful to humans and other animals through food chain transfer or direct ingestion [2,3].

The high emission of industrial wastes has produced numerous industrial wastelands around the world. Numerous metallurgical wastelands pose significant hazards to the health of the exposed population. For example, there are more than 450,000 sites contaminated with toxic chemicals in the United States (U.S.) [4]. Recent statistics released by the European Environment Information and Observation Network for soil (EIONET-SOIL) indicate that there are approximately 340,000 identified contaminated sites among the European countries surveyed, and the main contaminant categories are PTEs, contributing to approximately 35% of soil contamination [5]. By 2011, 2095 contaminated sites had been identified within 47 developing countries, and PTEs are the leading primary exposure [6].

Under environmental supervision by the Chinese government, numerous low-technology township enterprises, such as smelting factories, metal plating workshops, and chemical plants, have been shut down, and many industrial abandoned wastelands have resulted, which are mainly polluted by PTEs. These rural industrial abandoned wastelands not only encroach on large land resources but also bring about high levels of soil pollution, land degradation, and other detrimental effects. The remediation of PTEs contaminated sites is gaining considerable significance. Conventional technologies for site remediation are based on physical, chemical, and biological methods [7]. However, these traditional technologies could entail large costs or cause secondary pollution. Therefore, a comprehensive approach is urgently required to address this challenge [8,9]. Soil pollution assessment has been used as a diagnostic tool before making rational remediation strategies. The systematical quantitative evaluation of PTEs contamination contributes to the understanding of the actual pollution situation. The most commonly cited assessment indices in environmental studies include the integrated pollution load index (PLI), Nemerow pollution index (NPI), geo-accumulation index (I_geo_), potential ecological risk index (PERI), risk assessment code (RAC), enrichment factors (EF), and mean probable effect level quotient (m-P-Q) [10]. These methods are widely used to evaluate the heavy metal pollution in farming soils [11], anthropogenic soils [12,13], and sediments [14,15]. However, there are few studies examining the potential environmental risks of rural abandoned industrial wastelands in China, which are a troublesome type of point source contamination due to their higher remediation cost and limited funding [16,17].

To update the comprehensive understanding on the PTEs pollution of rural abandoned wastelands in North China, we chose a typical rural wasteland in southeast Baoding where rural industrial enterprises are densely distributed, and we evaluated the pollution levels and eco-risk by multiple assessment methods. The primary objectives of this study were: (1) to determine the spatial distributions and total concentrations of arsenic (As), cadmium (Cd), chromium (Cr), copper (Cu), lead (Pb), and zinc (Zn) in the rural industrial abandoned site in North China; (2) to identify anthropogenic contamination of heavy metal in topsoil using the PLI and I_geo_; (3) the RAC and a health risk assessment (HRA) were used to estimate the environmental risk and human exposure risk to PTEs in a typical rural wasteland in North China. The findings will be particularly useful for risk prevention and the ecological reclamation of industrial waste sites in rural North China.

## 2. Materials and Methods

### 2.1. Study Area

**Figure 1:**
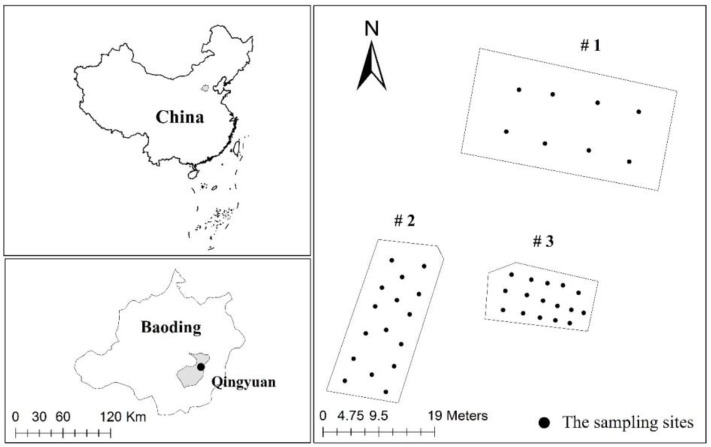
Sampling sites in a township metallurgy factory of Baoding, North China.

The study sites are in a suburb with an abandoned factory in southern Baoding, Hebei province, China, where small non-ferrous metallurgy enterprises are densely distributed. Three sites were investigated: site #1, site #2, and site #3 (shown in Figure 1). Based on a previous field survey, site #1 was in a natural area never used for industrial activities and waste depositions. Site #2 was abandoned for almost 20 years and characterized by the presence of slag from lead pyrometallurgy. Site #3 was abandoned for eight years and characterized by the presence of waste residue of zinc hydrometallurgy. The study area has a temperate continental monsoon climate with four distinct seasons; the annual dominant wind directions in Baoding are northeast/southwest (NE/SW), and its perennial average wind speed generally ranges between 4 and 6 m/s. The annual average precipitation is 550 mm, and the annual mean temperature is 12 °C. The main sources of soil pollution are metallurgy, chemical plants, electroplating, textile, and paper industries.

### 2.2. Soil Sampling and Chemical Analysis

Eight top soil samples (0–20 cm) from site #1 and 16 top soil samples from both sites #2 and #3 were collected using the random point method. These samples were placed in self-locking polyethylene bags and transferred to the laboratory. The soil samples were air-dried, ground, and passed through a 2 mm nylon sieve to remove roots, debris, glass, stones, and other impurities. Next, all samples were subjected to physico-chemical analyses. Soil pH was measured in a 1:2.5 soil:water suspension. Cation exchange capacity (CEC) was determined in triplicate by BaCl_2_ exchange followed by compulsive MgSO_4_ exchange. Total nitrogen (TN) was measured by the Kjeldahl procedure. Organic matter (OM) concentration was determined by the Walkley-Black method. Particle-size distribution was measured using the dry sieving method to determine the sand, silt, and clay percentages. Then, the undersize soil samples were passed through a plastic sieve with a mesh aperture of 0.154 mm. Each time, 0.1 g of soil was poured into a polytetrafluoroethylene jar and digested with a guarantee reagent mixture HF-HClO_4_-HNO_3_ in an automatic digestion instrument (LabTech Digi Block ST36, Beijing, China). The digested solution was cooled, filtered, and diluted to 25 mL. The PTE concentrations (As, Cd, Cr, Cu, Pb, and Zn) were determined by an axial view inductively coupled plasma-atomic emission spectrometer (AX ICP-AES, SPECTRO Analytical Instruments GmbH/SPECTRO ARCOS EOP). A procedural blank and a standard reference material GBW07401 (GSS-1, China National Center for Standard Materials) were included for quality assurance and quality control (QA/QC) (one blank and one standard for ten samples). The modified Community Bureau of Reference (BCR) sequential extraction procedure was used for PTE form analysis of all the soil samples. The four extracted fractions of PTEs were defined as the water/acid soluble fraction (F1), reducible fraction (F2), oxidizable fraction (F3), and residual fraction (F4) [1,18,19,20].

### 2.3. Risk Assessment of PTEs

#### 2.3.1. PLI

The integrated PLI reveals the overall pollution status of a sample. The PLI can be calculated from (PI_1_ × PI_2_ × PI_3_ × … × PI*_n_*) 1/*n*. The pollution index (PI) is defined as PI = C*_i_*/C_0*i*_, where C*_i_* is the concentration of the *i*th element in soil samples (mg/kg) and C_0*i*_ is its corresponding reference concentration (mg/kg). The values of C_0*i*_ used in this article are the national first-level standard values provided in GB15618-1995 [21,22]. PLI is classified into seven levels: background concentration (PLI = 0), no pollution (0 < PLI ≤ 1), no-to-moderate pollution (1 < PLI ≤ 2), moderate pollution (2 < PLI ≤ 3), moderate-to-high pollution (3 < PLI ≤ 4), high pollution (4 < PLI ≤ 5), or very high pollution (PLI > 5) [23].

#### 2.3.2. I_geo_

I_geo_ is an geochemical parameter to distinguish the influences of natural geological processes and human activities on soil PTEs. It can be calculated as I_geo_ = log_2_ (C*_n_*/1.5B*_n_*), where C*_n_* is the measured concentration of the element (mg/kg), and B*_n_* is the background value of the element (mg/kg). The constant factor of 1.5 in the equation indicates very small anthropogenic influence on the contents of investigated PTEs in the natural environment [24,25]. In this study, the background values of Chinese Hebei province were chosen as the geochemical reference. Pollution grades of I_geo_ are given as follows: no pollution (I_geo_ ≤ 0), no-to-moderate pollution (0 < I_geo_ ≤ 1), moderate pollution (1 < I_geo_ ≤ 2), moderate-to-heavy pollution (2 < I_geo_ ≤ 3), heavy pollution (3 < I_geo_ ≤ 4), heavy-to-extreme pollution (4 < I_geo_ ≤ 5), or extreme pollution (I_geo_ > 5) [26].

#### 2.3.3. RAC

RAC assesses the availability of metals in a solution by applying a scale to the percentage of PTEs present in the water/acid-soluble fraction (F1) [27,28]. Five levels of RAC were proposed: no risk (<1%, NR), low risk (1–10%, LR), medium risk (11–30%, MR), high risk (31–50%, HR), and very high risk (>50%, VHR) [29].

#### 2.3.4. Human HRA

The HRA of topsoil quantitatively reveals both carcinogenic and non-carcinogenic risks of the three exposure pathways (ingestion, dermal contact, and inhalation) to humans [30]. The average daily doses (ADDs) (mg/kg day) of potentially toxic metals via ingestion (ADD*_ing_*), dermal contact (ADD*_dermal_*), and inhalation (ADD*_inh_*) for both adults and children were estimated as follows:

ADD*_ing_* = 10^−6^ × C*_soil_* × (*Ing*R × *EF* × *ED*)/(*BW* × *AT*) (1)

ADD*_inh_* = C*_soil_* × (*Inh*R × *EF* × *ED*)/(*PEF* × *BW* × *AT*) (2)

ADD*_dermal_* = 10^−6^ × C*_soil_* × (*SA* × *AF* × *ABS* × *EF* × *ED*)/(*BW* × *AT*) (3)

The detailed explanation of the exposure parameters and values used to estimate the risks [31,32] are given in Table 1 [21].

The non-carcinogenic effects of PTE were assessed using the hazard quotient (HQ). The HQ is the ratio of the ADD of a PTE to its reference dose (RfD) [18] for the same exposure pathway. The RfD is the maximum daily dose of a PTE from a specific exposure pathway (shown in Table 2) that is believed not to cause an appreciable risk of deleterious effects to sensitive individuals during their lifetime. Hazard index (HI) has been developed to assess the overall potential for non-cancer effects caused by PTEs. The risk (RI) is regarded as the probability of developing any type of cancer throughout an entire lifetime of an individual due to exposure to a potential carcinogen. Slope factor (SF) is the probability of cancer development at the per unit exposure level of mg/(kg·day). The HI and RI [32] are calculated as follows:

(4) $$\mathrm{HI}=\sum HQ_{i}=\sum \frac{ADI_{i}}{RfD_{i}}$$

(5) $$\mathrm{Risk}\left(\mathrm{RI}\right)=\sum ADI_{i}\times SF_{i}$$

HQ or HI ≤ 1 indicates that non-carcinogenic risks are unlikely even for sensitive populations, whereas the potential for adverse effects may be a concern when HQ or HI > 1 [32]. As, Cd, and Cr were identified as human carcinogens by the International Agency for Research on Cancer (IARC) [33,34,35,36]. In addition, due to the lack of SF for Pb, Cu, and Zn, only the RI for As, Cd, and Cr were estimated [37,38]. The carcinogenic risk to human health from soil can be negligible (RI < 10^−6^), acceptable or tolerable (1 × 10^−6^ < RI < 1 × 10^−4^), and high (RI > 1 × 10^−4^).

### 2.4. Statistical Procedures

The experimental data were analyzed using a one-way ANOVA (SPSS 16.0). The Turkey’s test was used to test the difference between various soil physico-chemical factors, taking *p* < 0.05 as significant in the top soils of three abandoned sites.

## 3. Results and Discussion

### 3.1. Soil Properties

#### 3.1.1. Physico-Chemical Properties of Top Soil

On the basis of previous research, the pH, OM content, nitrogen content, and particle size distribution of the three sites are shown in Table 3 [20]. These results suggest that the topsoil is weakly alkaline soil at site #1, neutral soil at site #2, and acidic soil at site #3, which is related to the different production technologies of waste residue. The soil C/N ratio is 8.75 (<15) at site #1, indicating the easier organic nitrogen absorption of soil. However, the soil C/N ratios are 83.10 and 62.43 (much higher than 30) at sites #2 and #3, respectively, indicating that N-fertilizer is hardly available in these soils. The result of soil particle size analysis shows that the percentage of clay particles (grain size < 50 μm) in three abandoned sites was between 3% and 10%, which indicates their soil type is sand loam.

#### 3.1.2. PTEs Concentrations in the Top Soil of Three Rural Abandoned Sites

The statistics of six PTEs in top soils are presented in Table 4. In this research, we focus on explaining and analyzing soil PTEs pollution in the typical rural industrial wasteland in North China. It can be obversed that the content of Cr at site #1 and #2 are under the background value (BV) in Hebei province. The average content of four PTEs (Cr, Cu, Pb, and Zn) at site #1 does not exceed the Secondary National Standard of China (G-II). Except Cr, the average concentrations of the other five PTEs at site #2 all exceed the G-II, while the average concentrations of PTEs at site #3 all exceed the G-II. The maximum concentrations of the six tested PTEs at sites #1, #2, and #3 all exceed the soil background values of Hebei Province and the first-level standard of the Chinese national environment soil quality (G-I) [22].

**Figure 2:**
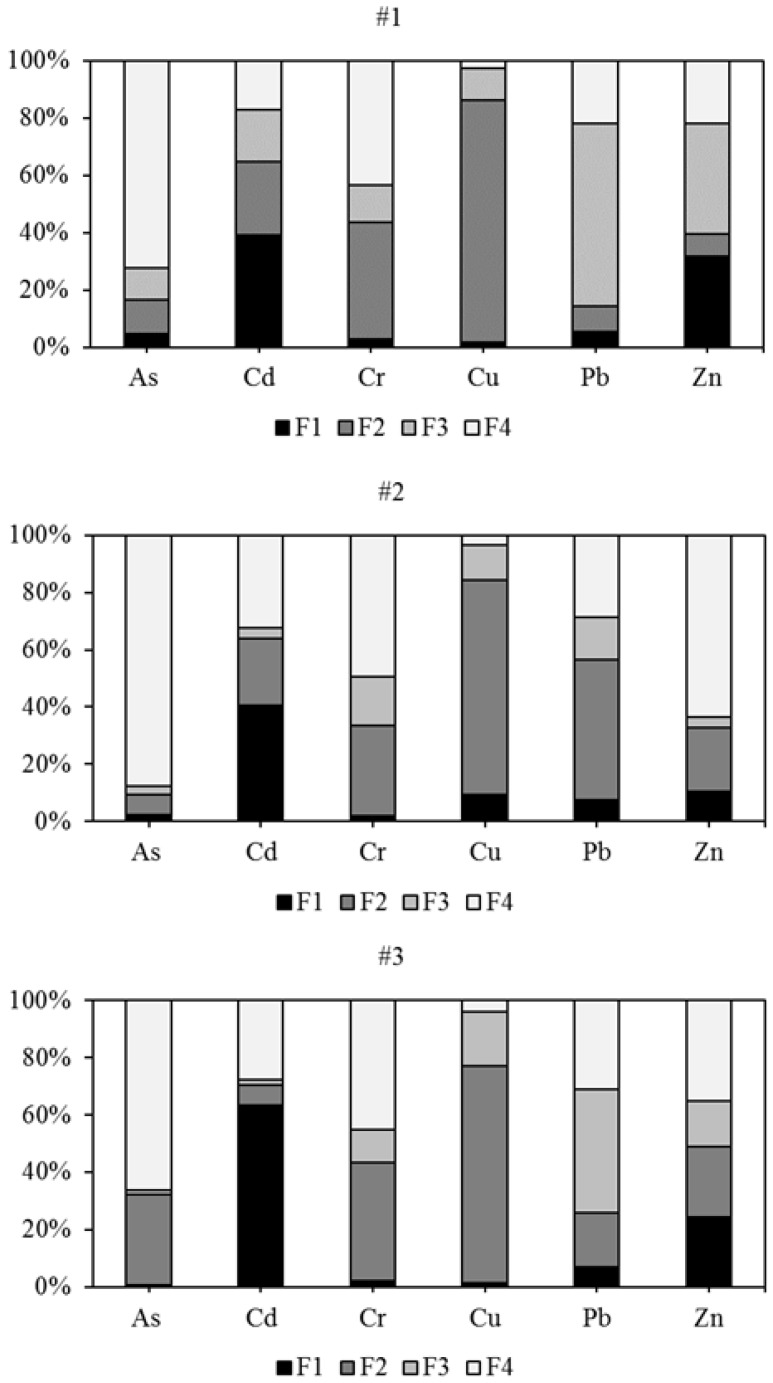
The speciation distributions of PTEs in top soils from three sites in North China. (F1, water/acid-soluble fraction; F2, reducible fraction; F3, oxidizable fraction; F4, residual fraction; Total, total metal concentration. The sample sites are: #1, #2, and #3).

The toxicity and mobility of PTEs were not only related to their total concentrations, but also their chemical speciation, their binding state, the metal properties, environmental factors, and soil properties like pH, OM content, and soil type [39]. The F1 of PTEs refers to exchangeable PTEs, which can be easily desorbed and migrated with water through soils and are sensitive to changes in soil pH. The F2 referred to metal associated with Fe and Mn oxides that was reducible, which might be released when subjected to more reducing conditions. The F3 referred to metal bound to OM that might be released under oxidizing conditions. The F4 is determined to be a stable fraction, which may be held in a primary and secondary mineral crystal structure and could not be affected by the environment [40]. Different fractions of PTEs in the soils have different biological effectiveness. The order of the bioavailability of metal fractions was F1 > F2 > F3 > F4. To determine the bioavailability of PTEs in the soils, the modified BCR was conducted, and the results are shown in Figure 2. For Cd and Zn, the F1 at sites #2 and #3 was much higher than that at site #1 because of the very high anthropogenic discharge concentrations, which indicates higher leaching toxicity and potential harm to the eco-environment. Generally, PTEs in F1 are bound to carbonates in the weakest strength and could be absorbed by the biota directly. In general, anthropogenic Cd and Zn are preferentially associated with carbonate minerals because of their similar ionic radius [41]. Cd ions or Zn ions could readily substitute for Ca ions in the carbonate-bound fraction [42]. Therefore, the higher proportions of Cd and Zn in the F1 might lead to deeper downward migration. In contrast, the elements of Pb at the three sites were mainly associated with the reducible forms (F2) and (F3), indicating relatively less mobility and bioavailability and a minor hazard to the eco-environment. The dominant phase of As and Cr in the top soils of all three sites was the residual fraction (Figure 2), which is bound in the mineral lattice. The availability of As, Cr, and Zn from the residual fraction is scarce; therefore, they are expected to be less harmful to biota. Cu in the top soils of three sites and the element of Pb at site #2 were dominated by the reducible fraction, indicating these two elements’ strong association with Fe/Mn oxides, from which Cu could be released into the water column under reduced conditions.

The dominant phase of Pb in the top soils of sites #1 and #3 was F3, indicating its integration with OM and lower leaching rate under prevailing environment conditions. However, the dominant phase of Pb in the top soil of site #2 was F2, indicating the anthropogenic Pb was mainly accumulated in the oxide fraction. This result is consistent with the findings indicating that Fe and Mn hydrous oxides have a strong adsorption capacity for Pb ions in soils and have an important effect in reducing its mobility in edatope [43].

### 3.2. Pollution Risk Assessment of a Rural Industrial Wasteland

#### 3.2.1. Contamination Indices (PLI and I_geo_)

**Figure 3:**
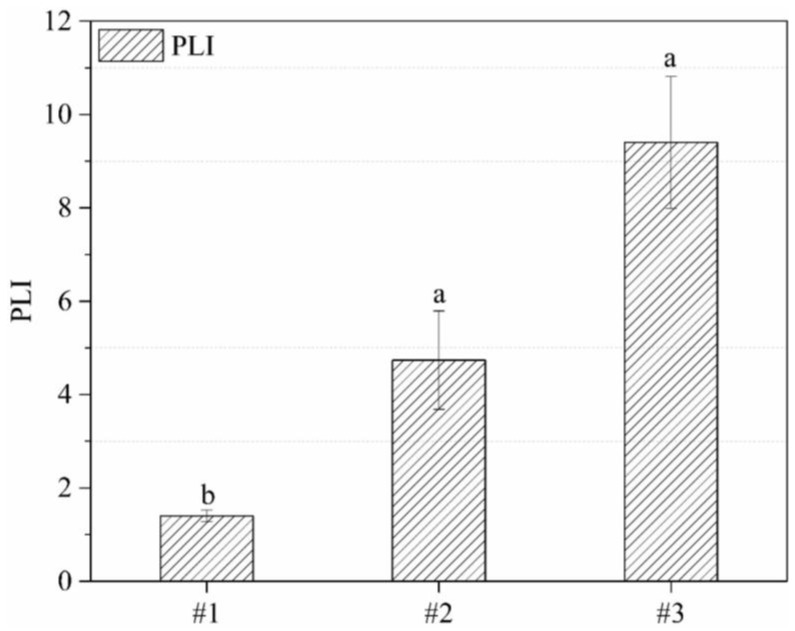
Difference of pollution load index (PLI ± SD) and statistical test (*p* < 0.05) between three abandoned sites. Bars marked with same letter are not significantly different at *p* = 0.05.

The PLI is proposed as a standardized system for assessing the overall pollution status that permits a comparison of pollution levels between different sites. It was first used for quantifying the estuarine quality in the simplest way [44] and is generally used for sediments assessment [45,46] and soil pollution impacts [47]. In this study, the PLI was computed to quantify the comprehensive toxicity status of three rural waste sites. The mean PLIs of the PTEs in the top soils of sites #1, #2, and #3 are shown in Figure 3.

The average PLI for site #1 was slightly higher than 1, suggesting that site #1 was basically unpolluted to moderately polluted by the tested trace metal(loid) elements. The average PLI for site #2 was less than 5, indicating the top soils of site #2 were highly polluted by lead smelting slag. However, the average PLI for site #3 substantially exceeded 5, indicating the top soils of site #3 were seriously polluted by the Zn residue dumping. The different pollution levels of the three sites demonstrated by the PLI relate to the metal concentration levels and different pollution sources.

**Figure 4:**
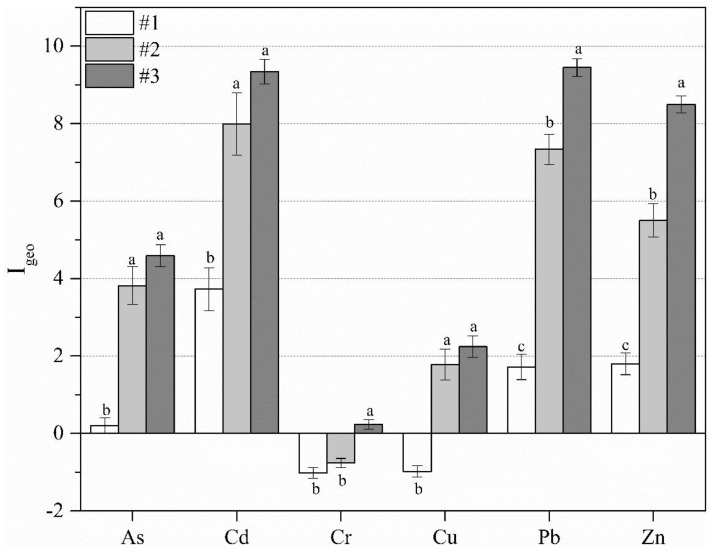
Difference of geo-accumulation index (I_geo_ ± SD) and statistical test (*p* < 0.05) between three abandoned sites. Bars marked with same letter are not significantly different at *p* = 0.05.

I_geo_ considers the human factors, geochemical background values, and the impact of diagenesis on background values, which directly reflect the degree of PTEs and the metal enrichment in soil. As shown in Figure 4, at site #1, the results of I_geo_ indicate no pollution by Cr or Cu, low As pollution, partial median pollution by Pb and Zn, and partial serious Cd pollution. These results indicate that Cd, Pb, and Zn concentrations are heavily affected by anthropogenic inputs, surface runoff, and dust from nearby contamination sources, while Cr and Cu were mainly of geochemical origin. At site #2, the mean I_geo_ of six PTEs indicate no Cr pollution, partial median Cu pollution, as well as partial serious-to-severe pollution by four PTEs (Cd > Pb > Zn > As > Cu). At site #3, the mean I_geo_ of four PTEs is greater than 4 (Pb > Cd > Zn > As > Cu), indicating significantly high pollution levels; the mean I_geo_ of Cu is 2.02, and the mean I_geo_ of Cr is slightly higher than zero (Figure 4). The results of I_geo_ indicate that site #1 was practically unpolluted to moderately polluted, while sites #2 and #3 were both heavily polluted by anthropogenic inputs of Pb, Zn, Cd, and As, and Cr was mainly of geochemical origin [48]. The results show little difference between sites #2 and #3, owing to their similar raw material (copper lead-zinc ore) for metallurgy. However, the OM and pH were significantly different (*p* ≤ 0.05) between sites #2 and #3. The results indicated that Cd at sites #3 showed geo-accumulation in an acid condition. Fine grain sediments and more OM facilitate the accumulation of more PTE contents. Therefore, sites #2 and #3 with higher OM may increase the Pb accumulation [49].

#### 3.2.2. RAC

**Figure 5:**
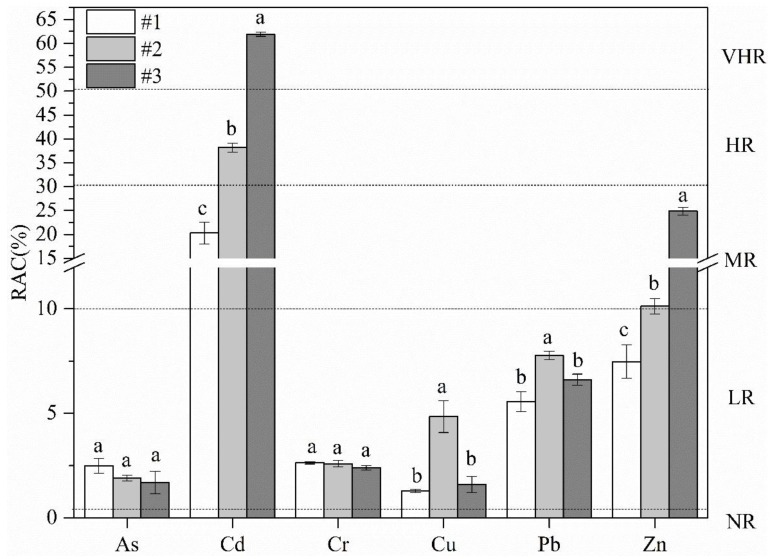
Difference of risk assessment code (RAC ± SD) and statistical test (*p* < 0.05) between three abandoned sites. VHR = very high risk; HR = high risk; MR = medium risk; LR = low risk; NR = no risk. Bars marked with same letter are not significantly different at *p* = 0.05.

Generally, PTEs in the F1 are bound to carbonates by relatively weak electrostatic interactions and could be more rapidly absorbed by biota [50]. Therefore, the percentage of PTEs introduced by anthropogenic activities in F1 could indicate a potential migration risk for PTEs to the eco-environment [51]. The RACs of six PTE elements at sites #1, #2, and #3 are displayed in Figure 5.

The RACs for As, Cr, Cu, and Pb among the rural industrial abandoned sites indicate a low risk (LR) to the environment, which was not in accordance with other evaluation methods (I_geo_). Although the total concentrations of As and Pb showed heavy contamination according to the I_geo_, the lower RACs of As and Pb came from their low level of association with the F1. These results indicated that the release of As and Pb in the top soil of two industrial wastelands were unlikely under prevailing environmental conditions, and so these elements showed a low toxicity to the surrounding area. However, the highest RAC value of Cd at site #3 was more than 50%, suggesting a very high environmental risk to the ecosystem. The RAC of Zn at site #3 was the largest among three sites, with a value of 24.69%, indicating a medium risk to the environment. The RAC method has been reported in previous studies, and similar evaluation results for I_geo_ and RAC have been reported by several authors [52,53]. The ecological risks of As and Pb were higher when they were calculated by the I_geo_ based on the total contents of pollutants. The low background levels of As and Pb (shown in Table 2) directly contributed to the relatively high degrees of enrichment obtained for these elements using the I_geo_ versus the results of the RAC method, which considered their relatively low exchangeable fraction (F1). Compared with site #1 (control plot), the RAC values of PTEs at sites #2 and #3 significantly increased (*p*  <  0.05) for Cd and Zn, suggesting that there was serious contamination of Cd and Zn in the top soils due to historical industrial activities. PTEs introduced by anthropogenic activities in the Chinese rural abandoned waste sites carry a potential risk to the ecological environment in terms of speciation (especially for Cd and Zn) and thus deserve more attention.

#### 3.2.3. Human HRA

The assessment results of the health risk of six toxic PTEs exposures at the three sites are shown in Table 5.

At site #1, the mean HQs and HIs for both children and adults via any of the three exposure pathways are less than 1, which indicates a non-carcinogenic risk or negligible health risk. The RIs for both children and adults are lower than 10^−6^, indicating that the carcinogenic risk of PTEs in the soils at site #1 could be neglected. These findings support the results from site investigation and PLI assessments.

At site #2, the HQ*_ing_* and HI values of As and Pb are both greater than 1, which indicate the main non-carcinogenic pollutants for children and adults are As and Pb. Both the HQs and HIs for children are larger than those for adults, suggesting that children are more susceptible to non-carcinogenic risk. The HQs and HIs of Cd, Cr, Cu, and Zn are lower than 1, indicating there would be no adverse health effects for children and adults via the three exposure pathways. The HQs of five PTEs (As, Cr, Cu, Pb, and Zn) for children decrease by the exposure pathway in the following order: ingestion > inhalation > dermal contact, and the HQs of Cd change in the following order: ingestion > dermal > inhalation contact, which is the same as in other studies [54]. These results are consistent with previous findings, which indicated children’s potential health risks are mainly caused by the direct oral ingestion of contaminated soils rather than dermal contact and inhalation [32,55]. The HI values of PTEs decrease in the following order: Pb > As > Cd > Zn > Cr > Cu for both children and adults (Table 5). Due to the lack of carcinogenic slope factors for Pb, Cu, and Zn, only the carcinogenic risks of As, Cd, and Cr were estimated (Table 5). Similarly, RI values are also higher for children than those for adults. However, almost all the RI values of PTEs for children and adults were lower than 10^−6^, except for As, where the RI value for children was slightly higher than 10^-6^, indicating its slight carcinogenic risk at site #2. In addition, the toxicity of Cr is mainly dependent on its valence state, and the Cr (VI) is more toxic to biota than Cr (III) [56]. The total mean concentration and water-soluble fraction of Cr (shown in Table 4 and Figure 2) in the top soils at site #2 were at low levels. Therefore, the actual carcinogenic hazard of Cr for children may be overestimated due to the higher value of carcinogenic slope factors and actual low total concentrations for Cr in the studied areas [57].

At site #3, the HQs of As and Pb are larger than 1, which indicated there were adverse health effects on both children and adults via ingestion. For the six PTEs, the contributions to HI (total non-carcinogenic risk) are the highest for HQ*_ing_*, with 99.98% for children and 99.67% for adults, suggesting that ingestion is the main exposure pathway that threatens human health. This conclusion is consistent with other studies [32,58]. The HI values of As and Pb for children and adults are higher than 1, indicating the main non-carcinogenic pollutants are As and Pb. Similarly, HIs and RIs were also higher for children than those for adults at site #3, indicating a relatively high hazard to children’s health. The RI values of As and Cr for children are slightly higher than 10^−6^, suggesting the carcinogenic risks of As and Cr at site #3 can not be neglected, and children are faced with more health risks in daily life than adults via the unconscious ingestion pathway. However, the RI value of Cr may also be overestimated in the top soils at site #3 because the total mean concentration of Cr was used, which was lower than the second-class National Soil Environment Standard (pH < 6.5).

Although sites #2 and #3 were two different kinds of metallurgy slag polluted land, both were similar in their HRA results for their total concentration of PTEs. Previous studies have reported that the most dangerous exposure route of non-cancer risk is direct oral ingestion [59,60]. Chronic exposure of Pb can damage the nervous, skeletal, circulatory, enzymatic, endocrine, and immune systems [61]. Exposure to As occurs generally in the form of either arsenite (As(III)) or arsenate (As(VI)) and the increased cancer risk is attributed to (As(III)) rather than the less toxic (As(VI)) [62]. Although the HI values of Cd were lower than 1 at sites #2 and #3, the health risk of Cd could not be overlooked, as the excessive intake of Cd may lead to chronic diseases, such as pulmonary adenocarcinomas, bone fractures, kidney dysfunction, and hypertension [63]. Additionally, Zn in soils of studied rural wastelands may also pose a public health risk, considering its extremely high concentrations. It has been documented in many studies that children have higher non-carcinogenic risks than adults through three pathways of these PTEs, indicating that children are more vulnerable than adults to toxic PTEs exposure because they have higher respiration rates per unit body weight, unconscious and unsafe hand-to-mouth activities in contaminated soils, and immature detoxification capabilities [64]. Therefore, children should be carefully nursed and must be kept away from contaminated soil environments to avoid exposure. Further research is necessary to explain the reasons for the carcinogenic risk caused mainly by As in the studied Chinese rural metallurgical wasteland. Despite some inconsistencies among the HRA, I_geo_, and RAC results, the evaluations using different approaches can provide some valuable recommendations for government regulators and environmental workers to discern priority control PTEs, develop an economically restoration strategy, and manage the rural abandoned industrial wasteland effectively in rural China [65].

## 4. Conclusions

A typical metallurgical wasteland in a rural area of North China was chosen to represent the high pollution and widely distributed industrial abandoned wastelands in vast rural areas of China. The concentrations, pollution levels, and health risk assessment of PTEs in the topsoil at three sites of an abandoned rural factory in Baoding were thoroughly investigated. In general, the results of the PLI showed that the abandoned industry wasteland (sites #2 and #3) was heavily polluted by toxic trace elements compared with site #1. Furthermore, the results of the I_geo_ suggested that As, Cd, Pb, and Zn are key toxic elements and priority control PTEs at both sites #2 and #3. For site #1, there were almost no health risks for adults because the HQs, HIs, and RIs were all lower than the threshold levels. However, the HQ*_ing_* and HI values of As and Pb for children and adults were above the threshold levels, indicating that the health of children and adults may be affected by As and Pb after long-term exposure to sites #2 and #3. The main exposure pathway for both children and adults was ingestion, followed by dermal contact and inhalation. The RI values of As and Cr for children were slightly higher than 10^−6^, suggesting that carcinogenic risks at site #3 could not be neglected. Thus, risk recognition and further enhanced natural revegatation measures are needed to reduce exposure risks for susceptive groups in hazardous environments. Based on the site risk assessments in this research, safe landfill of high concentration slags combined with in-situ phytostabilization by indigenous species may be an economical and ecological solution for rural industrial abandoned sites remediation in North China in the future.

## Figures and Tables

**Table 1:** Reference values of parameters for health risk assessment (HRA) of potential toxic elements (PTEs) pollution [21].

Factor	Definition	Unit	Value	
C*_soil_*	PTE Concentration in Soil	mg/kg	Children	Adults
*Ing*R	Ingestion rate of soil	mg/day	200	100
*EF*	Exposure frequency	days/year	350	350
*ED*	Exposure duration	years	6	24
*BW*	Body weight of the exposed individual	kg	15	55.9
*AT*	Average time	days	365ED	365ED
*Inh*R	Inhalation rate of soil	m^3^/day	7.63	12.8
*PEF*	Particle emission factor	m^3^/kg	1.36 × 10^9^	1.36 × 10^9^
*SA*	Exposed skin surface area	cm^2^	1600	4350
*AF*	Skin adherence factor	mg/(cm·day)	0.2	0.7
*ABS*	Dermal absorption factor	unitless	0.001	0.001

**Table 2:** Reference doses (RfD) and slope factors (SF) for HRA of PTEs pollution [38]. Subscripts: ing = ingestion; inh = inhalation; derm = dermal contact.

Elements	RfD_ing_ (mg/(kg·d))	RfD_inh_ mg/(kg·d)	RdD_derm_ mg/(kg·d)	SF_inh_ (mg/(kg·d))^−1^	SF_ing_ (mg/(kg·d))^−1^	SF_dermal_ (mg/(kg·d))^−1^
As	3.00 × 10^−4^	1.50 × 10^−5^	1.23 × 10^−4^	1.51 × 10	1.50	3.66
Cd	1.00 × 10^−3^	1.00 × 10^−3^	1.00 × 10^−5^	6.30		
Cr	3.00 × 10^−3^	2.86 × 10^−5^	6.00 × 10^−5^	4.20 × 10		
Cu	4.00 × 10^−2^	4.02 × 10^−2^	1.20 × 10^−2^			
Pb	3.50 × 10^−3^	3.52 × 10^−3^	5.25 × 10^−4^			
Zn	3.00 × 10^−1^	3.00 × 10^−1^	6.00 × 10^−2^			

**Table 3:** Basic soil properties of the topsoil collected at three waste sites [20].

Parameter	#1	#2	#3
pH (1:2.5)	8.22 ± 0.03 a	7.54 ± 0.07 b	6.30 ± 0.06 c
CEC ^1^ (cmol/kg)	8.17 ± 0.18 c	12.18 ± 0.22 a	10.72 ± 0.51 b
Organic C (g/kg)	14.01 ± 1.24 c	24.93 ± 1.72 b	44.37 ± 1.53 a
Total N (%)	0.16 ± 0.03 a	0.03 ± 0.02b c	0.07 ± 0.04 ab
C/N	8.75	83.10	62.43
Gravel > 2 mm (%, *w*/*w*)	25.30 ± 1.00 b	31.23 ± 0.79 a	15.50 ± 0.44 c
Sand 2–0.05 mm (%, *w*/*w*)	67.00 ± 0.93 b	62.40 ± 0.55 c	76.30 ± 1.23 a
Silt-Clay < 0.05 mm (%, *w*/*w*)	4.93 ± 0.09 a	3.70 ± 0.12 b	3.20 ± 0.06 c

^1^ Cation Exchange Capacity. Mean values with rows followed by same letter are not significantly different according to Turkey’s test (*p* < 0.05).

**Table 4:** Descriptive statistics of PTE concentrations (mg/kg) in top soils of the three abandoned sites.

Site		As	Cd	Cr	Cu	Pb	Zn
#1	Min.	9.40	0.23	29.50	21.10	40.00	179.00
	Max.	27.00	8.14	61.60	35.90	203.00	684.00
	Median	14.20	2.31	51.85	29.35	122.00	512.35
	Mean	15.76	2.81	47.51	29.01	114.90	473.10
	Std.D	5.88	2.32	12.26	5.43	59.47	165.17
	Skewness	1.07	2.03	−0.60	−0.16	0.24	−0.76
	Kurtosis	0.74	5.27	−1.37	−1.42	−1.18	0.09
#2	Min.	32.40	3.75	36.20	75.00	768.10	1137.70
	Max.	1695.10	621.10	72.10	1402.10	26,288.30	11,461.40
	Median	80.10	10.90	58.05	208.50	2725.35	2219.60
	Mean	269.91	63.40	58.47	301.69	5496.16	4486.42
	Std.D	435.45	153.99	8.94	333.21	7153.04	4182.15
	Skewness	2.72	3.60	−0.68	2.77	2.20	1.02
	Kurtosis	8.00	13.47	1.37	8.41	4.52	−0.80
#3	Min.	105.00	9.65	65.70	106.80	6071.00	6071.00
	Max.	1464.00	164.00	758.00	956.00	49,973.00	39,353.10
	Median	329.50	106.00	118.00	429.45	26,143.50	23,412.50
	Mean	433.47	95.38	162.67	454.79	24,503.25	21,332.69
	Std.D	365.41	46.19	163.08	226.73	10,508.13	9100.86
	Skewness	1.61	−0.50	3.65	0.77	0.34	−0.01
	Kurtosis	3.11	−0.63	14.02	0.66	1.64	−0.61
	^1^ BV	8.70	0.075	63.90	53.50	20.00	78.40
	^2^ G-I	11.2	0.097	61.00	22.60	26.00	74.20

^1^ BV means background values of Hebei Province. ^2^ G-I means the first-level standard of the Chinese national environment soil quality.

**Table 5:** Health risks of PTEs in soils on rural abandoned metallurgy lands in north China. HQ = hazard quotient; HI = hazard index.

**Site #1**	**PTEs**	**As**	**Cd**	**Cr**	**Cu**	**Pb**	**Zn**
HQ*_ing_*	Children	4.53 × 10^−1^	3.12 × 10^−2^	1.27 × 10^−1^	5.52 × 10^−3^	3.09 × 10^−1^	1.34 × 10^−2^
	Adults	6.08 × 10^−2^	4.19 × 10^−3^	1.70 × 10^−2^	7.40 × 10^−4^	4.15 × 10^−2^	1.80 × 10^−3^
HQ*_inh_*	Children	2.54 × 10^−4^	8.76 × 10^−7^	3.73 × 10^−4^	1.54 × 10^−7^	8.63 × 10^−6^	3.76 × 10^−7^
	Adults	1.14 × 10^−4^	3.94 × 10^−7^	1.68 × 10^−4^	6.93 × 10^−8^	3.88 × 10^−6^	1.69 × 10^−7^
HQ*_der_*	Children	1.64 × 10^−6^	4.62 × 10^−6^	9.37 × 10^−6^	2.72 × 10^−8^	3.05 × 10^−6^	9.91 × 10^−8^
	Adults	5.80 × 10^−5^	1.64 × 10^−4^	3.32 × 10^−4^	9.65 × 10^−7^	1.08 × 10^−4^	3.52 × 10^−6^
HI	Children	4.54 × 10^−1^	3.12 × 10^−2^	1.27 × 10^−1^	5.52 × 10^−3^	3.09 × 10^−1^	1.34 × 10^−2^
	Adults	6.10 × 10^−2^	4.35 × 10^−3^	1.75 × 10^−2^	7.41 × 10^−4^	4.16 × 10^−2^	1.80 × 10^−3^
Risk	Children	5.76 × 10^−8^	5.52 × 10^−9^	4.47 × 10^−7^			
	Adults	2.59 × 10^−8^	2.48 × 10^−9^	2.01 × 10^−7^			
**Site #2**							
HQ*_ing_*	Children	1.01 × 10	8.61 × 10^−1^	1.37 × 10^−1^	7.36 × 10^−2^	1.62 × 10	1.39 × 10^−1^
	Adults	1.35	1.15 × 10^−1^	1.83 × 10^−2^	9.87 × 10^−3^	2.18	1.86 × 10^−2^
HQ*_inh_*	Children	5.67 × 10^−3^	2.41 × 10^−5^	4.02 × 10^−4^	2.05 × 10^−6^	4.52 × 10^−4^	3.89 × 10^−6^
	Adults	2.55 × 10^−3^	1.09 × 10^−5^	1.81 × 10^−4^	9.25 × 10^−7^	2.04 × 10^−4^	1.75 × 10^−6^
HQ*_der_*	Children	3.65 × 10^−5^	1.27 × 10^−4^	1.01 × 10^−5^	3.63 × 10^−7^	1.60 × 10^−4^	1.03 × 10^−6^
	Adults	1.29 × 10^−3^	4.52 × 10^−3^	3.59 × 10^−4^	1.29 × 10^−5^	5.67 × 10^−3^	3.64 × 10^−5^
HI	Children	1.01 × 10	8.61 × 10^−1^	1.37 × 10^−1^	7.36 × 10^−2^	1.62 × 10	1.39 × 10^−1^
	Adults	1.36	1.20 × 10^−1^	1.89 × 10^−2^	9.89 × 10^−3^	2.18	1.86 × 10^−2^
Risk	Children	1.28 × 10^−6^	1.52 × 10^−7^	4.83 × 10^−7^			
	Adults	5.78 × 10^−7^	6.85 × 10^−8^	2.18 × 10^−7^			
**Site #3**							
HQ*_ing_*	Children	1.30 × 10	7.60 × 10^−1^	5.13 × 10^−1^	9.11 × 10^−2^	5.47 × 10	5.55 × 10^−1^
	Adults	1.75	1.02 × 10^−1^	6.89 × 10^−2^	1.22 × 10^−2^	7.34	7.45 × 10^−2^
HQ*_inh_*	Children	7.30 × 10^−3^	2.13 × 10^−5^	1.51 × 10^−3^	2.54 × 10^−6^	1.53 × 10^−3^	1.56 × 10^−5^
	Adults	3.29 × 10^−3^	9.60 × 10^−6^	6.80 × 10^−4^	1.14 × 10^−6^	6.87 × 10^−4^	7.01 × 10^−6^
HQ*_der_*	Children	4.70 × 10^−5^	1.13 × 10^−4^	3.80 × 10^−5^	4.50 × 10^−7^	5.40 × 10^−4^	4.11 × 10^−6^
	Adults	1.67 × 10^−3^	3.99 × 10^−3^	1.35 × 10^−3^	1.59 × 10^−5^	1.91 × 10^−2^	1.46 × 10^−4^
HI	Children	1.30 × 10	7.60 × 10^−1^	5.15 × 10^−1^	9.11 × 10^−2^	5.47 × 10	5.55 × 10^−1^
	Adults	1.75	1.06 × 10^−1^	7.09 × 10^−2^	1.22 × 10^−2^	7.36	7.46 × 10^−2^
Risk	Children	1.65 × 10^−6^	1.34 × 10^−7^	1.81 × 10^−6^			
	Adults	7.44 × 10^−7^	6.05 × 10^−8^	8.17 × 10^−7^			
